# Impulsiveness as potential moderators of the relation between social media dependence and eating disorders risk

**DOI:** 10.1186/s40359-022-00830-8

**Published:** 2022-05-08

**Authors:** Zhonghua He, Weili Yang

**Affiliations:** 1 School of Journalism and New Media, Xi’an Jiaotong University, No. 28, Xianning West Road, Xi’an, 710049 Shaanxi People’s Republic of China; 2 School of Public Health, Shaanxi University of Chinese Medicine, Xi’an, People’s Republic of China

**Keywords:** Social media dependence, Eating disorders risk, Non-planned impulsiveness, Moderating role

## Abstract

**Background:**

Social media dependence (SMD) and eating disorders (ED) risk are often thought to be inextricably linked. Because social media dependence often precedes an ED, predicts poor outcome, and persists even after recovery from an ED, it is important to examine whether certain factors have the ability to potentially attenuate or intensify SMD’s effect on eating disorders.

**Methods:**

In the current study, we examined one possible moderating factor: impulsiveness. 767 undergraduates (mean age = 18.78 years, SD = 1.20) in Shaanxi province of China, completed anonymous questionnaires regarding social media dependence, eating disorders, impulsiveness.

**Results:**

Revealed that non-planned impulsiveness (NPI) moderated the relation between SMD and ED risk. Individuals who were low in SMD and who reported low levels of NPI reported much lower levels of ED risk than those with low SMD and high NPI. However, Individuals who were high in SMD and who reported low levels of NPI reported much higher levels of ED risk than those with high SMD and high NPI. Contrary to our hypotheses, Motor impulsiveness and cognitive impulsiveness did not emerge as moderators of the relation between SMD and ED risk.

**Conclusions:**

Results provide growing support that factors that interact with SMD can lessen or aggravate SMD’s effect on ED risk. These findings can be beneficial to our understanding of how and when social media dependence impacts undergraduates’ eating disorders risk.

## Introduction

As Internet technology continues to develop, social media such as Weibo, WeChat, QQ, Tik Tok and so on have become an indispensable part of life. According to the latest released official report from the China Internet Network Information Center (CINIC), there are almost 0.751 billion netizens in China, accounting for 1/5 of the netizens globally [1]. Of these, most social media users constitute young adults at the age of 18–24 years. The top three most popular used social medias were Wechat (utilization ratio: 85.5%), QQ (utilization ratio: 67.8%), and Sina Weibo (utilization ratio: 37.1%) [1]. As early contact and wide users of social media, college students' dependent behavior of social media is particularly prominent [2]. Its widespread use has increased the ease of interpersonal communication between individuals, and socialization processes; yet, problematic use of social media has become prevalent among a large proportion of users and led to significant behavioral and psychological problems [3]. Problematic social media use has repercussions on users’ social, psychological, and personal lives [4]. When individuals are so engaged in social media that they feel distressed when they are unable to use it, such misuse is widely referred to as social media dependence (SMD) [5, 6].

Online social media behaviors (e.g., viewing and commenting) have been significantly correlated with a drive for thinness among undergraduate students [7]. Eating disorders (ED) risk, especially among young adults, have become a worldwide concern [8]. Stanghellini proposed [9] that persons with ED experience their own body first and foremost as an object being looked at by another, rather than from a first-person perspective. They developed and validated a new self-reported questionnaire named IDEA (IDentity and eating disorders), which represents a multidimensional, brief, versatile, easy-to-perform instrument [9]. IDEA has been reported to be specifically associated with the core features of ED psychopathology and to show good internal consistency, valid incremental validity as compared with other scales devoted to the measure of eating disorders psychopathology (e.g. eating disorders Examination Questionnaire and eating disorders Inventory, EDI-1) [9–11].

### The relation between social media dependence and eating disorders risk among college students

Social media dependence has been associated with an increased risk of eating disorders especially among young adults who are easier to access internet and spend most of their time for social networking [12]. Associations between social media use and ED pathology was found [7, 13–16]. Time spent on Facebook appears to be associated with body image dissatisfaction and eating disorders pathology, with the relationship between Facebook and ED stronger compared to viewing ‘Barbie’ type models on television and magazines [13]. Social media can create a climate of social comparison and preoccupation with thinness and beauty, which can pose risks for emotional problems, such as depression and social anxiety. With the widespread use of social media, this behavior triggers the extreme pursuit of thinness and beauty among youths. In face of beautiful images on social media, young adults are more likely to resent their physical image [17]. Those who are more dissatisfied with their appearance are more likely to suffer from depression, eating disorders risks [18]. Previous studies have found, young adults who use more Facebook are more dissatisfied with their appearance [19]. Besides, Facebook use may contribute to disordered eating by maintaining risk (i.e. weight/shape concerns and state anxiety) for eating pathology compared to an alternate internet activity [20]. In many ways, social media dependence and eating disorders tendency are closely intertwined [14, 20].

The use of social media is most common among college students than other adults [8]. Young adults primarily use social media for communication, entertainment, and professional development, making it indispensable for college students [9]. Life habits are usually formed during young adulthood [12]. College students are easy to form habits in life and behavior, and more easily influenced by their peers, with strong plasticity [4]. More time spent on Facebook relates to more frequent body and weight comparisons, more attention to others’ physical appearances, and more negative feelings about their own bodies [21]. College students are, thus, at risk of disordered eating attitudes owing to the elevated mental and physical demands of higher education [22].

### Impulsiveness as a moderator

Does the relation between social media dependence and eating disorders risk have something to do with impulsivity? Impulsiveness is defined as action without good planning and with little consideration of the consequences. Self-control can be defined as the choice of a larger, more delayed reinforcer over a smaller, less delayed reinforcer, and impulsiveness as the opposite [23]. Acting impulsively is an action based on the impulse to express a desire [24]. Acting impulsively also means acting without thinking about the action first, a behavior that wants to get immediate feedback from the environment, or impatient behavior to delay its desire. The cause of a person behaving impulsively is a case related to attention and active behavior, so there is no definite cause. According to experts, there are many factors that encourage a person to behave impulsively, such as the nature that tends to hyperactive, temperamental, environment influences. The message that ‘thinness is beauty’ circulated in social media environments, attracts expanding research attention and growing interests. Impulsivity was regarded as a multi-dimensional construct. Historically, research on impulsivity in eating disorders has been primarily focused on individuals with bulimic-spectrum disorders (i.e., those involving binge eating and/or purging behaviors, including bulimia nervosa, binge eating disorders, purgative anorexia nervosa and binge eating/purging subtype of anorexia nervosa) [25].

Although social media use, such as Facebook, may lead to eating disorders risk [26], it is possible that not all people are equally influenced by its effects. Consequently, it is important to examine variables that may moderate the relationship between social media dependence and negative outcomes. It’s worth noting that restrictive anorexia nervosa patients (AN-R) reported less impulsiveness scores than controls [27, 28]. In general, maladaptive aspects of perfectionism (e.g., perseverance behaviours), the opposite of several traits of impulsiveness (e.g., lack of planning and perseverance), is well-established as the risk factors for the relationship between social media environment and eating disorders [29, 30]. They found support for the moderation hypothesis, with girls high on both perfectionism and body dissatisfaction exhibiting the highest levels of eating disorders symptoms. Thus, impulsiveness may be an important factor that impacts the relation between social media dependence and eating disorders risk [31]. Therefore, the relationship between the social media environment and eating disorders was not only related to the level of social media dependence but to impulsivity and self-control. The higher the degree of social media dependence and the planned self-control, the more likely individuals are to be influenced by the social media thin-ideal content and adhere to unhealthy eating behaviors and body image dissatisfaction. On the contrary, for the individuals with high impulse-type and poor self-control, although they are influenced by social media thin-ideal content, it is difficult for them to stick to their idea and actions [27].

### The current study

In the present study, we tested moderator models of the relation between social media dependence and eating disorders risk. To summarize, the current study examined the relationship between social media dependence and eating disorders risk. Firstly, we expected that social media dependence would be a risk factor for eating disorders tendency, with high social media dependence predicting high eating disorders tendency. Secondly, we further explored whether this association would be moderated by impulsiveness, and expected that the direct effect would be stronger for individuals with low impulsiveness than for those with high impulsiveness. Given that low self-control may buffer people from the negative impact of social media environment, we expect that high impulse-type might instead serve as a protective factor for the effect of high level of social media dependence on identity and eating disorders risk [27].

## Methods

### Participants and procedure

The sample consisted of 767 undergraduates. Among the participants, the average age was 18.78 (SD = 1.20) with 552 females (71.97%) and 215 males (28.03%). Students were recruited through limited optional courses open to the university. University students form suitable samples because they are more likely to be frequent users of social media, including use of a variety of computer mediated- communication functions [32].

This study is part of a project approved and authorized by by biomedical ethics committee of the Medical Department, Xi’an Jiaotong University in accordance with the ethical standards identified and in compliance with the relevant guidelines and regulations. Inclusion criteria were those with no reported significant medical impairments or physical/mental problems. These were verified via questionnaire items, stating that the female undergraduates have never been diagnosed with eating disorders at the time of joining the study, nor have they undergone pharmacological treatment and/or participated in individual or group psychotherapy owing to mental problems (e.g., learning disabilities, diagnosed eating disorders, and anxiety disorders). The participants answered survey questions about their social media dependence, eating disorders tendency, impulsiveness and demographic information. There was no reward for answering survey questions. After indicating an interest in the study, they were sent survey questions via scanning the WeChat QR code. Their informed consents were then obtained online by clinking the questionnaire link. Participants could save their answers on the website, allowing them to return to the survey in case they accidentally disconnected or did not complete it at one time.

### Measures

#### Social media dependence

This variable was measured through the 23-item the Mobile Social Media Dependence questionnaire [33]. Participants rated how true each statement was for them on a 5-point scale ranging from 1 = “completely not true” to 5 = “completely true.” The scale contains items such as “Playing mobile social media is my daily habit,” and “Browsing mobile social media during self-study or class has affected my learning efficiency”. This assessment tool measures five different dimensions using five subscales, including social gain, salience, compulsivity, conflict, withdrawal. This questionnaire exhibited satisfactory psychometric qualities, and Cronbach’s alpha of five subscales ranged from 0.75 to 0.80.

#### Impulsiveness

The Chinese Version of the Barratt Impulsiveness Scale 11th Version [34] is a 30-item self-report questionnaire designed to assess characteristics of impulsiveness. The measure contains three subscales: (a) cognitive impulsiveness (attention and cognitive instability), (b) motor impulsiveness (motor impulsiveness and perseverance), and (c) non-planned impulsiveness (self-control and cognitive complexity). Scoring of items on the Likert-type scale range from 1 (never) to 5 (always), in which higher scores reflect greater impulsiveness. Cronbach’s alpha values were 0.72 for cognitive impulsiveness subscale, 0.86 for motor impulsiveness subscale, 0.81 for non-planned impulsiveness subscale, respectively.

#### Eating disorders tendency

The Chinese Version of IDEA Questionnaire consists of 23 items divided into four subscales: (1) feeling oneself through the gaze of the other and defining oneself through the evaluation of the other (GEO); (2) feeling oneself through objective measures (OM); (3) feeling extraneous from one’s own body (EB); and (4) feeling oneself through starvation (S) [9, 10]. IDEA has been reported to show good internal consistency (Cronbach’s alpha value = 0.95) [35]. The whole scale of the IDEA had an internal consistency coefficient of 0.94 for the present sample. Cronbach’s alpha values were 0.89 for GEO subscale, 0.91 for OM subscale, 0.80 for EB subscale, 0.80 for S subscale, respectively.

### Analytic strategy

To test the hypothesized moderator models, hierarchical multiple regression was utilized in the prediction of eating disorders tendency (IDEA) [36]. In Step 1, social media dependence and the moderator of impulsiveness (e.g., Motor impulsiveness) were entered as main effects. In Step 2, the two-way interaction of social media dependence and the moderator (e.g., Social Media Dependence × Motor impulsiveness) was entered. Interaction terms were created by multiplying together the centered, continuous social media dependence variable and the centered, continuous moderator as recommended by Frazier, Tix, and Barron [37]. The nature of the significant interactions was assessed via simple slope analyses [38, 39].

## Result

### Descriptive statistics

Means and standard deviations for the study variables and their correlations are presented in Table 1. Correlations were basically as expected based on the literature, including positive correlations between eating disorders tendency (IDEA) and both social media dependence and impulsiveness and negative correlations between age and impulsiveness.

**Table 1:** Correlations among and means and standard deviations of the measured variables (N = 767)

	1	2	3	4	5	6	7	*M*	*SD*
1.Age	–							18.77	1.20
2.Gender	− .09*	–						1.72	.45
3.IDEA	.06	− .10**	–					55.70	13.11
4.SMD	.03	.11**	.50***	–				15.85	2.68
5.MI	− .03	.02	.40***	.22***	–			24.76	5.81
6.CI	− .11**	.09*	.11**	.17***	.19***	–		27.20	4.06
7.NPI	− .15***	.03	.09*	.18***	.20***	.71***	–	24.42	5.03

*IDEA* Identity and eating disorders, *SMD* Social Media Dependence, *MI* Motor Impulsiveness, *CI* Cognitive Impulsiveness, *NPI* Non-planned Impulsiveness

*p < .05. **p < .01. ***p < .001

### Social media dependence × impulsiveness interactions predicting eating disorders tendency

Altogether, three hierarchical multiple regressions were performed involving the interaction of social media dependence and impulsiveness in the prediction of eating disorders tendency, the results of which are displayed in Table 2. The SMD × NPI interaction was significant in predicting Identity and eating disorders scores (*t* (760) = − 2.72, *p* = 0.007), but the other two interaction terms were not.

**Table 2:** Hierarchical multiple regression analyses of the interaction of social media dependence and impulsiveness moderators in the prediction of Identity and eating disorders (IDEA) after controlling for age and gender

Step and predictors	B	SE B	*β*	*t (dfs)*	*p*
*Step1*					
Social media dependence	2.17	.15	.45	14.92(4761)	< .001
Motor impulsiveness	.67	.07	.30	9.99(4761)	< .001
*Step 2*					
SMD × MI	.02	.02	.04	1.28(5760)	.200
*Step1*					
Social media dependence	0.51	.09	.48	5.95(4761)	< .001
Cognitive impulsiveness	.19	.10	.06	1.90(4761)	.058
*Step 2*					
SMD × CI	.00	.00	0.01	0.16(5760)	.873
*Step1*					
Social media dependence	2.46	.15	.51	15.97(4761)	< .001
Non-planned impulsiveness	.05	.08	.02	.58(4761)	.565
*Step 2*					
SMD × NPI	− .06	.02	− .08	− 2.72(5760)	.007

*SMD* Social media dependence, *MI* Motor impulsiveness, *CI* Cognitive impulsiveness, *NPI* Non-planned impulsiveness

**Fig. 1:**
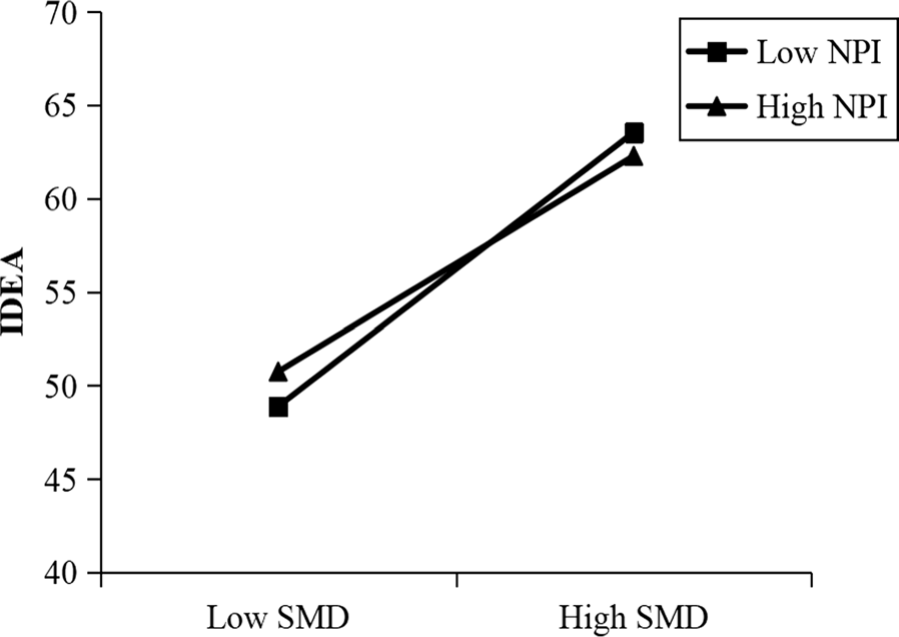
The interaction of social media dependence and non-planned impulsiveness with IDEA scores as the dependent variable. The moderating effect is graphed for two levels of NPI: 1 standard deviation above the mean and 1 standard deviation below the mean

As depicted in Fig. 1, those with high levels of social media dependence who engaged in low levels of non-planning impulsiveness had higher levels of eating disorders tendency than those with high levels of social media dependence and high non-planning impulsiveness, suggesting that high levels of non-planning impulsiveness may buffer the effect of high social media dependence on eating disorders tendency. On the contrary, those with low levels of social media dependence who engaged in high levels of non-planning impulsiveness had higher levels of eating disorders tendency than those with low levels of social media dependence and low non-planning impulsiveness. Simple slope tests showed that for low non-planning impulsiveness individuals [40], higher social media dependence was associated with eating disorders tendency, *B*_simple_ = 0.61, *p* < 0.001. However, for high non-planning impulsiveness individuals, the effect of social media dependence on eating disorders tendency was weaker, *B*_simple_ = 0.45, *p* < 0.001.

Overall, the effect of social media dependence on eating disorders tendency was moderated by non-planning impulsiveness. For low SMD, low NPI served as a protective factor to reduce the potential risk of social media dependence for eating disorders tendency. However, for high SMD, low NPI served instead as a negative factor to increase the potential risk of social media dependence for eating disorders tendency.

## Discussion

In the present study, we examined how the relation between social media dependence and eating disorders risk may vary depending on levels (high vs. low) of various impulsiveness in a sample of college students. Our hypothesis that impulsiveness would moderate this relation was partially supported. We found that non-planning impulsiveness (but not motor- or cognitive impulsiveness) moderated the relation between social media dependence and eating disorders risk, as measured by the IDEA.

The present study extended previous research efforts by examining the association between social media dependence and ED risk. According to some literature, an association between time spent on Facebook and ED pathology was found [13]. The integrated cognitive-behavioral theory of ED identifies a feedback loop whereby exposure to body-related stimuli activates and reinforces an over-concern with one’s own body, which in turn reactivates attentional biases toward body-related stimuli [41]. The frequency of this feedback loop serves to generate or maintain ED and this process could possibly explain the finding of a relationship between frequent Facebook use and ED risk [41]. Accordingly, it may be the case that frequent exposure to thin-ideal content on social media reinforces one’s own body related concerns, eliciting cognitive biases that lead one to selectively attend to thin-ideal content on Facebook [13]. An extreme drive for thinness is associated with the development and maintenance of eating disorders, according to many theories [41].

As expected, individuals who were high social media dependence and self-report low non-planning impulsiveness had significantly higher eating disorders risk than those with high social media dependence and high levels of non-planning impulsiveness. Planning ability (PA) is a key aspect of cognitive functioning and requires individuals to identify and organize the necessary steps to achieve a goal [42]. The non-planning impulsiveness score also assesses lack of prior planning or goal orientation [43]. Given that planning is an essential cognitive process of executive functions and is considered as one of the most important brain functions [44], it is not surprising that the combination of high social media dependence and high levels of PA was associated with heightened eating disorders risk.

The interesting thing is that among individuals with low level of social media dependence, low non-planning impulsiveness instead seems to serve as a protective factor for eating disorders risk. Social media use has become an important aspect of individuals’ social lives [45]. Social identity theory states that how one perceives his or her own identity is affected by the environment and those that individual is around [46, 47]. As the dependence on social media decreases, they can define themselves in those environments they live around to much more than ‘thinness is beauty’ circulated social media environments.

This study contributes to the existing literature by expanding our understanding of the relation between SMD and ED risk using more complex moderator model. However, the present study should be interpreted in light of three limitations. First, other factors such as self-esteem, personality could be influencing in relation between SMD and ED risk [48, 49]. In future research, these factors should be considered. The second limitation of this study that lies in the Chinese undergraduate sample population implies that the results of this study may not be widely generalized to other countries or cultures. Third, the time of social media use or the type of application was not measured either, which affect us to get a more detailed and clear relational model [50].

Despite these limitations, this research has important practical implications. First, the recruitment strategy was an additional strength. By sampling from a non-clinical sample other than an eating disorders clinic, we were able to examine a broad range of eating disorders risk, contributing to the early prevention on youths from getting ED. Second, given that the moderating effect of non-planning impulsiveness in the relation between SMD and ED risk, we should pay more attention to college students with low no-planning impulsiveness (high planning ability) and high social media dependence in terms of the prevention of eating disorders risk. Last but not least, it should be recognized that social media use acts as a double-edged sword in our daily lives [39], it makes our life more convenient, but it is affecting our health at the same time.

## Data Availability

The data used during the current study are available from the corresponding author on reasonable request.
